# Report of Deaths in the City of Buffalo for the Month of February, 1862

**Published:** 1862-04

**Authors:** Sandford Eastman

**Affiliations:** Health Physician


*Report of Deaths in the City of Buffalo for the month of February,* 1862.

Abscess 2, Accident 3, Albuminuria 1, Apoplexy, cerebral 1, Bronchitis 1, Cancer 2, Cancer of the Stomach 1, Cholera Infantum 1, Consumption 23, Convulsions 8, Croup 7, Cyanosis 3, Debility 1, Delirium Tremens 1, Diabetes Mellitus 1, Disease of the Brain 1, Disease of the Heart 1, Disease of the Liver 1, Diptheria 3, Dropsy 1, Epilepsy 2, Fever Puerperal 1, Fever Scarlet 12, Fever Typhoid 6, Hœmorrhage from Bladder 1, Inflammation of the Bowels 3, Inflammation of the Brain 3, Inflammation and Meninges 4, Inflammation Larnyx 1, Inflammation of the Lungs 15, Inflammation of the Lungs and Pleura 1, Inflammation of the Spine 1, Inflammation of the Stomach 1, Intemperance 2, Measles 1, Old Age 5, Paralysis 1, Premature Birth 2, Rheumatism 1, Stricture of Æsophagus 1, Suicide 1, Whooping Cough 1, Unknown 5. Deaths from Disease 132. Still Born 7. Total 139.

SANDFORD EASTMAN, Health Physician.

